# Exploring the Proteomic Profile of Soybean Bran: Unlocking the Potential for Improving Protein Quality and Quantity

**DOI:** 10.3390/plants12142704

**Published:** 2023-07-20

**Authors:** Mayla Daiane Corre Molinari, Renata Fuganti-Pagliarini, Yanbao Yu, Lilian Hasegawa Florentino, Liliane Marcia Mertz-Henning, Rayane Nunes Lima, Daniela Matias de Carvalho Bittencourt, Marcelo Oliveira Freire, Elibio Rech

**Affiliations:** 1 Arthur Bernardes Foundation, Embrapa Soybean, Londrina 86085-981, Brazil; mdcmolinari@gmail.com; 2 Piccola Scientific Consulting, Saskatoon, SK S7P 0B6, Canada; renatafuganti@gmail.com; 3 J. Craig Venter Institute, Rockville, MD 20850, USA; yayu@jcvi.org; 4 Embrapa Genetic Resources and Biotechnology, National Institute of Science and Technology in Synthetic Biology, Distrito Federal 70770-917, Brazil; lilian.florentino@embrapa.br (L.H.F.); rayanenl@gmail.com (R.N.L.); daniela.bittencourt@embrapa.br (D.M.d.C.B.); 5 Embrapa Soybean, Londrina 86085-981, Brazil; liliane.henning@embrapa.br; 6 J. Craig Venter Institute, La Jolla, CA 92037, USA; mfreire@jcvi.org

**Keywords:** *Glycine max*, BRS 537, industrial processing, bioactive proteins, grain quality, LC–MS/MS, proteomic profile

## Abstract

Soybean is a rich source of vegetal protein for both animal and human consumption. Despite the high levels of protein in soybean seeds, industrial processing to obtain soybean bran significantly decreases the final protein content of the byproducts. To overcome this problem, cultivars with higher protein contents must be developed. However, selecting the target proteins is difficult because of the lack of information on the proteome profile of soybean bran. Therefore, this study obtained the comparative proteomic profiles of both natural coatless seeds and defatted bran from an elite tropical-soybean cultivar. Thus, their extracts were characterized using LC–MS/MS and a total of 550 proteins were identified. Among these, 526 proteins were detected in coatless seeds and 319 proteins in defatted bran. Moreover, a total of 139 proteins were identified as presenting different levels of content in coatless seeds and defatted bran. Among them, only 46 were retained after the seed processing. These proteins were clustered in several important metabolic pathways, such as amino-acid biosynthesis, sugar biosynthesis, and antioxidant activity, meaning that they could act as targets for bioactive products or genome editing to improve protein quality and quantity in soybean grains. These findings can enhance our understanding regarding protein robustness for both soybean crops and the commercial bran improvement because target proteins must remain intact after processing and must be bioactive when overexpressed. Overall, the soybean bran proteomic profile was explored for the first time, providing a valuable catalogue of target proteins that can tolerate the industrial process.

## 1. Introduction

Soybean (*Glycine max*) is one of the most important commodities on the international market. In addition to its multiple uses in animal nutrition and human food consumption, this grain can be used in many industrial processes, as well as in medicine, owing to its health benefits [1,2,3,4,5,6]. Among the byproducts of soybean, bran is produced through standard soybean milling processes. It is commonly utilized in animal feed, as a fiber source for dairy and beef cattle, in pet food with reduced fat content, and as a supplement in animal feed [7,8].

Overall, soybean seeds have a high concentration of protein (40%) and are abundant in essential amino acids, such as phenylalanine, valine, tryptophan, threonine, lysine, leucine, and isoleucine, and sulfur-containing amino acids, such as methionine and cystine, which are essential for a balanced diet [9,10]. Although natural soybean grains are rich in proteins, most are degraded during soybean processing. This degradation occurs because of many factors, including the mechanical, thermal, and chemical steps involved in processing [11]. These processing factors influence protein properties and can modify their solubility and digestibility, thereby affecting their potential applications in the food industry. Thus, there are two challenges that hamper the development of soybean bran with a higher quantity and quality of proteins. The first one is industrial soybean processing, which involves high heating treatments, resulting in a significant loss of proteins due to denaturation [12]. The second one is that practical breeding progress increases seed protein but decreases yield. Given that seed protein contents have a negative genetic correlation with seed yield, other seed components such as oil and sucrose, along with interactions with environmental effects such as temperature during seed development [12,13,14]. To reduce these obstacles, the development of soybean cultivars with enhanced protein and amino acid content would further increase the crop’s economic value and would contribute to enriching the entire value chain, from farmers to processors to end-users [12].

To mitigate these losses, which decrease the economic value of soybean grains, many biotechnology strategies have focused on improving complex multigene traits, such as the overexpression of genes involved in improving the protein content in the grain [15]. However, to improve the soybean bran protein content, it is necessary to identify target proteins that must remain intact after processing and become bioactive when overexpressed by synthetic biology. If the overexpressed protein is not tolerant to processing, it is lost in the bran.

Currently, the most significant research on soybean seed proteomics includes the differential expression of the cotyledon, mature seed, seed coat, and seeds at different developmental stages, including conventional, transgenic, and mutated soybean cultivars [16,17,18,19,20,21,22,23,24,25]. Some of these studies have aimed at the quantitative analysis of proteomic profiles, comparing different stress conditions [6]. Nevertheless, the available literature on the soybean proteome is limited to data regarding unprocessed soybean seeds, and the studies do not represent the protein content available in by-products, such as bran, after industrial processes.

To overcome the lack of information on the protein remaining after soybean processing, we identified and compared the protein profiles of both natural coatless seeds and processed defatted bran from the elite soybean cultivar, BRS 537 (Embrapa, Brazil). The proteomic and biological pathway data described here provide knowledge on the molecular composition of elite tropical soybeans by focusing on proteins tolerant to industrial processing stages, aiming to allow researchers to develop innovative strategies to improve the nutritional quality of soybean products by providing a robust catalogue of target proteins.

In this work, we focused on identifying and comparing the protein profiles of natural coatless seeds and processed defatted bran from the elite soybean cultivar, BRS 537 (Embrapa, Brazil). Our intention is not only to contribute to the existing body of knowledge on the molecular composition of elite tropical soybeans, but also to highlight proteins that display resilience during processing stages. The proteome analysis of soybean bran after processing allowed the detection and quantification of anti-nutritional factors and allergenic proteins, which is vital to ensure food safety and proper labeling of allergens in food products. Lastly, the ultimate aim is to provide a robust catalogue of target proteins that could aid researchers in developing innovative strategies to improve the nutritional quality of soybean products.

## 2. Results

### 2.1. Comparative Proteomic Profile

**Figure 1:**
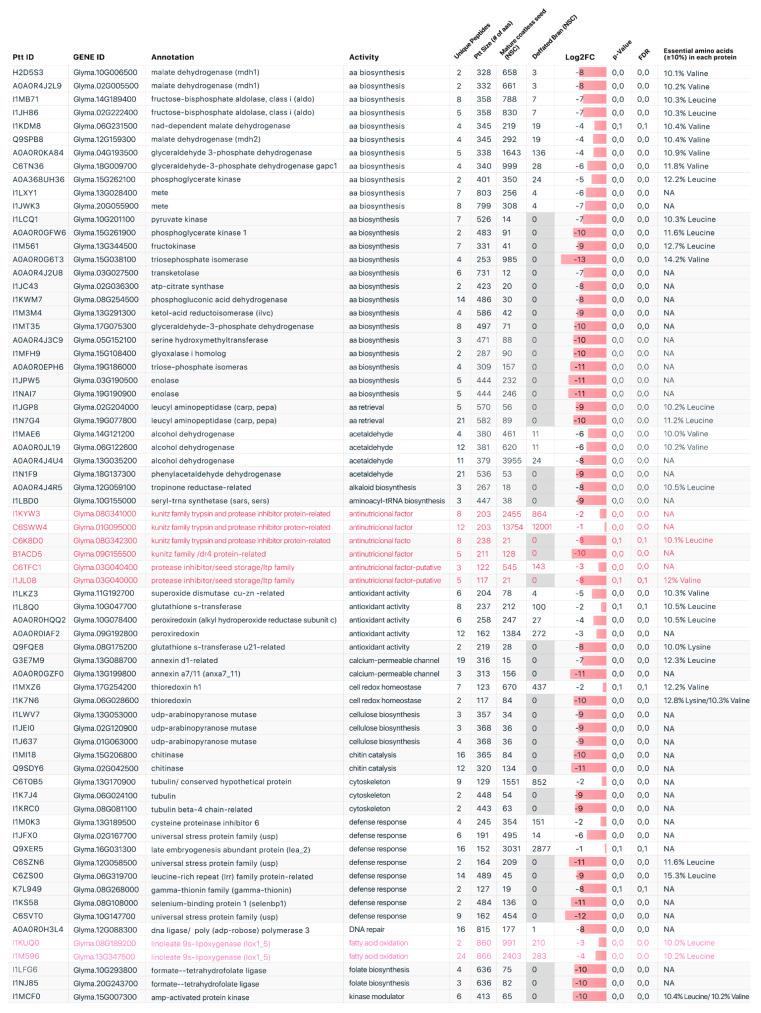

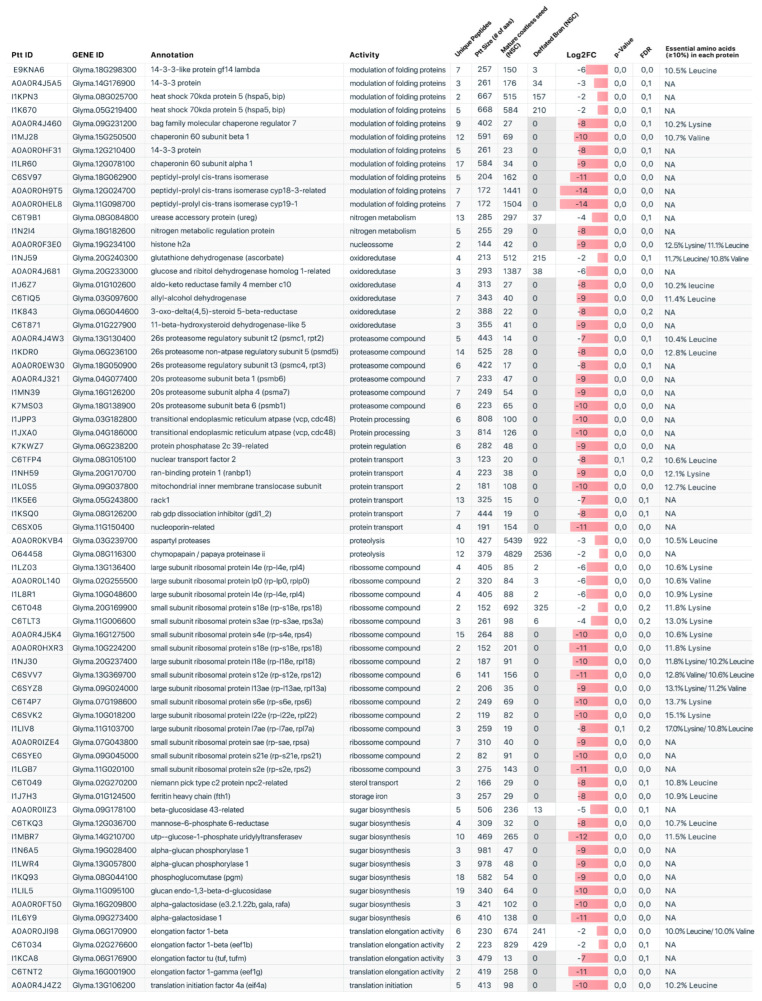
Differentially expressed proteins identified in coatless soybean seeds and defatted bran. Protein ID or UniProt is provided as well as gene ID, annotation, related activity, number of unique peptides, protein size (in number of amino acids), normalized spectrum counts, soybean coatless seed and defatted bran Log2FC, *p*-value, false-discovery rate, and percentage of essential amino acids (higher than 10%). Proteins classified as anti-nutritional factors are highlighted in red; lipoxygenases are marked in pink, and proteins present in coatless seed but that were completely lost in defatted bran are highlighted in gray. Legend: Ptt: protein; FDR: false-discovery rate; NA: not applicable; NSC: normalized spectrum counts.

**Figure 2:**
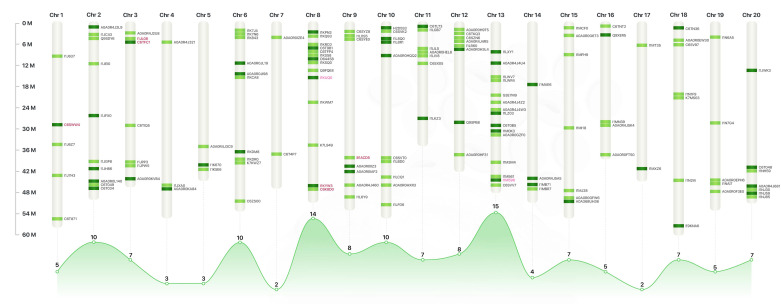
Chromosomal positions of all the genes that encode the 139 differentially expressed proteins. Dark green represents proteins completely retained in the defatted bran after soybean processing. Light green represents proteins lost in defatted bran. Proteins marked in red are anti-nutritional factors, and proteins marked in pink are lipoxygenases (LOX).

LC–MS/MS was used to analyze and identify the comparative proteomic profile from the elite soybean cultivar, BRS 537 (Embrapa, Brazil). A total of 550 proteins were identified. Among these, 526 proteins were detected in coatless seeds and 319 proteins in defatted bran. Moreover, a total of 139 proteins were identified as presenting different levels of content in coatless seeds and defatted bran (Figure 1). However, 93 proteins were completely lost in the bran after soybean processing and the 46 proteins retained after the seed treatment showed lower protein levels (Figure 1). The chromosomal positions of all the genes that encode the 139 differentially expressed proteins are indicated in Figure 2. Furthermore, the complete list of the 550 identified proteins along with their absolute counts in spectrum counts is shown in the Supplementary File S1.

Regarding the amino acid composition, of the 139 proteins with different contents, 64 were identified as rich in essential amino acids if, in their protein’s total amino acid composition, more than 10% comprised either valine, leucine, lysine, or a combination of these essential amino acids. It is important to note that essential amino acids with less than 10% of the total protein composition were not considered. Among those considered, 14 proteins showed more than 10% valine in their composition, 30 proteins contained more than 10% leucine, and 11 proteins featured more than 10% lysine. A combination of more than 10% leucine and valine was present in four proteins, 10% lysine and leucine were observed in three proteins, and 10% lysine and valine were noted in two proteins. In total, 34 proteins (53%) considered rich in essential amino acids were lost in the defatted bran, with 30 proteins (47%) retained after processing (Figure 1).

The cultivar’s genome was sequenced by Embrapa, and it is available at NCBI GenBank under access GCA_012273815.2 assembly (https://www.ncbi.nlm.nih.gov/assembly/GCA_012273815.2/ accessed on 29 May 2022). The novelty of this information is that most of the available soybean-protein databases, such as the Soybean Proteome Database (http://proteome.dc.affrc.go.jp/Soybean/ accessed on 29 May 2022), are limited to data from unprocessed soybean seeds and generally do not describe the protein content available in the processed bran.

### 2.2. Protein Functional Categories

The KEGG-analysis results identified 32 different pathways represented in the coatless seeds and defatted bran. Of these 32 pathways, 15 were lost after soybean processing, with no proteins remaining (amino acid retrieval, alkaloid biosynthesis, aminoacyl-tRNA biosynthesis, calcium-permeable channel, chitin catalysis, folate biosynthesis, kinase modulator, nucleosome, proteasome compound, protein processing, protein regulation, protein transport, sterol transport, storage iron, and translation initiation). For all the others 17, at least one protein remained in the defatted bran (Figure 1).

Applying the criterion of the identification of three or more proteins in a pathway for it to be considered enriched, the KEGG results pointed to 14 enriched pathways. Thus, 25 proteins were identified as involved in the amino acid biosynthesis pathway; 16 in the ribosome compound; 11 in the modulation of protein folding; 9 in sugar biosynthesis; 8 in defense response; 6 in oxidoreductase, proteasome compound, protein transport, and anti-nutritional factor (2 putative); 5 in antioxidant activity; 4 in translation-elongation factor and acetaldehyde; and 3 in cellulose biosynthesis and the cytoskeleton (Figure 1).

Two proteins were identified in the pathways involved in amino acid retrieval, the calcium-permeable channel, cell-redox homeostasis, chitin catalysis, fatty-acid oxidation, folate biosynthesis, nitrogen metabolism, protein processing, and proteolysis. Only one protein was identified in the following pathways: alkaloid biosynthesis, aminoacyl-RNA biosynthesis, DNA repair, kinase modulation, protein regulation, nucleosome, sterol transport, iron storage, and translation initiation (Figure 1). One important group of proteins for the human food industry identified in both the coatless seeds and the defatted bran was the lipoxygenases, despite the group’s undesirable flavor (2).

**Figure 3:**
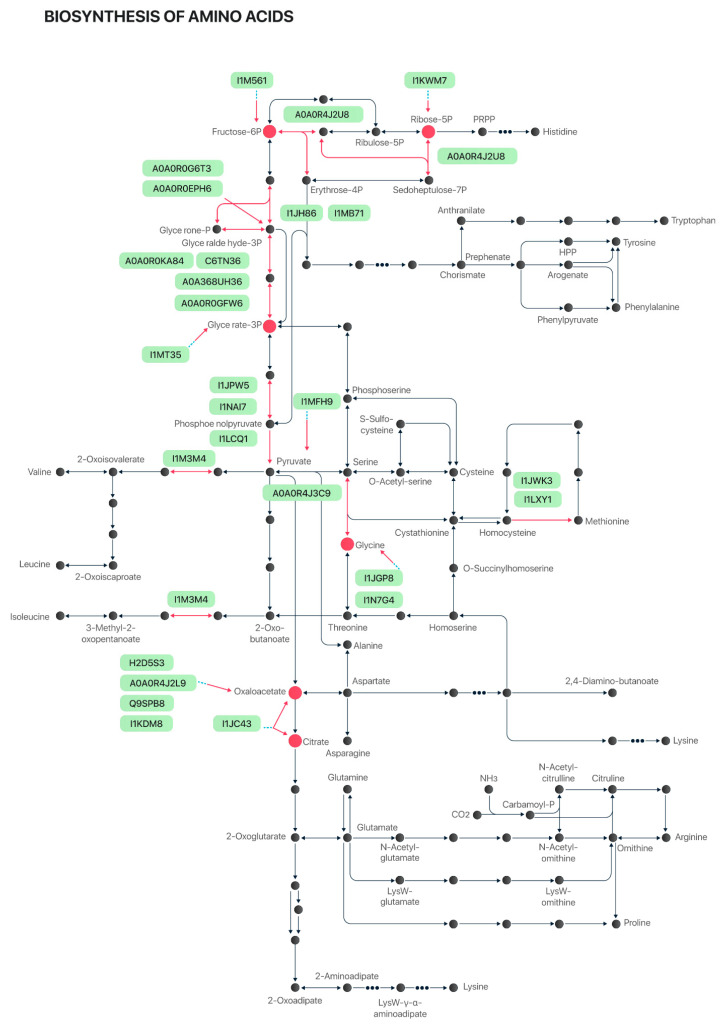
Amino-acid biosynthesis pathway. The 25 proteins differentially expressed are highlighted in light green and their respective metabolic-activity positions are indicated by red arrows and red dots.

To illustrate the complexity of the protein biosynthesis pathway, all 25 proteins identified either in the coatless seeds or in the defatted bran are combined in Figure 3. It is important to highlight that 15 of these 25 proteins were identified as rich in essential amino acids. In addition, 14 of the 25 proteins were lost in the defatted bran after industrial processing, and 6 of them were classified as rich in essential amino acids.

## 3. Discussion

Soybeans are an important driving force in the industrial economies of producing countries. Some data suggest that the international market for soybean-protein ingredients was valued at around USD 9.7 billion in 2018 and it is projected to reach USD 16.6 billion by 2026, at a compound annual growth rate (CAGR) of 6.9% [26]. These numbers explain why the industry is focused on developing cultivars with more sources of added value, such as higher protein content. However, the use of industrial processes to obtain soybean by-products can decrease protein and essential amino acid contents. Soybean bran is the by-product that remains after the outer layer of the soybean seed has been removed and the oil has been extracted. It is widely recognized for its high protein content and significant levels of dietary fiber, making it a promising ingredient for diverse food applications and a potential source of bioactive compounds. Therefore, analyzing the proteomic profile of soybean bran after processing provides valuable information about its nutritional and functional properties. This knowledge can help us find value-added applications for this agricultural by-product, thereby reducing waste and increasing the overall profitability of the soybean industry. Thus, researchers have focused on identifying key proteins and developing genetic engineering and synthetic-biology strategies to overcome the loss of protein content and nutritional quality during industrial food processes.

Soybean bran has emerged as an abundant and cost-effective source of essential amino acids. It comprises all nine essential amino acids in significant amounts, making it a valuable component in a balanced diet. The essential amino acids found in soybean bran include methionine, valine, threonine, phenylalanine, isoleucine, leucine, histidine, tryptophan, and lysine. In this study, more than 50% (26 out of 46) of the proteins maintained in the defatted bran were considered rich in essential amino acids, since more than 10% of their protein composition comprised valine, leucine, lysine, or a combination of these. The loss of these proteins in the defatted bran after processing is an industry concern, as these essential amino acids are directly related to the quality and quantity of the total protein content in soybean by-products. It is worth noting that even if not fully intact, proteins rich in essential amino acids enhance the quality of soy-based products. This is an important feature for industry animal feed as poor bran quality interferes with the maintenance, growth, and reproduction of animals, as well as the production of final products, such as meat, eggs, and milk [27].

In this study, although some of the proteins identified in coatless seeds and defatted bran did not present a composition rich in essential amino acids, they can play important roles in the metabolic pathways that are involved in the synthesis of proteins and essential amino acids, or even act on their targets (bioactive). This would be of interest in genetic engineering and the development of bioproducts (Figure 1).

In addition to the biosynthesis pathways of essential amino acids, other pathways are also shown in Figure 3. The metabolism of sulfur-containing amino acids, such as methionine and cysteine, has been connected to several crucial aspects of human and animal health and nutrition [27,28]. Methionine is an essential amino acid, and cysteine is considered a “conditional” essential amino acid since mammals can produce cysteine from methionine. However, mammals are unable to synthesize essential amino acids on their own and must obtain them from their diet [27], highlighting the importance of cultivating soybean varieties with higher protein content for animal feed and human supplementation.

In the animal feed industry, when properly processed for specific purposes, soybean grains and by-products can be used to feed all types of animals, including companion animals, domestic animals, poultry, swine, and aquatic life. Soybean by-products are rich in amino acids such as lysine, tryptophan, threonine, isoleucine, and valine, which are often lacking in cereal grains, such as corn and sorghum, both commonly used in poultry and swine feed. These amino acids are essential for ruminants and monogastric animals. However, soybeans also contain anti-nutritional factors, such as lectins, hemagglutinins, isoflavones, phytic acid, trypsin, and protease inhibitors, and this must be addressed to increase their nutritional value for the industry [29]. Soybean anti-nutritional factors can have significant implications for the nutritional value and utilization of soybeans. The impact of anti-nutritional factors in soybean consumers can vary depending on the species, age, and physiological status of the animals, as well as the level and duration of exposure to these compounds. Further research is needed to understand the complex interactions between anti-nutritional factors in soybeans and their effects on animal and human health. Efforts should focus on developing novel techniques to effectively reduce or eliminate anti-nutritional factors while preserving the nutritional integrity of soybean-based products. In this study, six proteins were identified as anti-nutritional factors, all of which are related to the Kunitz family of trypsin and protease inhibitors (Figure 1). The effect of soybean trypsin inhibitors on ruminants and monogastric animals has been extensively studied. Most of these reports show that the nutritional value of soybeans for ruminants and monogastric animals is limited by these anti-nutritional factors, which can interfere with feed intake and nutrient metabolism [30,31,32,33].

Soybean possessing high levels of protease inhibitors, particularly trypsin inhibitors, can negatively affect protein digestibility and amino acid availability. While heat processing can inactivate these protease inhibitors, excessive heat can also destroy other proteins, nutrients, and essential amino acids [29]. In this context, the identification of key proteins from these anti-nutritional families and the use of biotechnological tools to develop cultivars with lower contents of anti-nutritional factors are desired, as these would allow for better and more efficient use of the protein intake in the diet, making them promising approaches for the industry. It is important to emphasize that the expression of anti-nutritional factors is influenced by diverse elements. One previous study evaluated soybean genotypes and the expression of five anti-nutritional factors and two Kunitz trypsin inhibitors, demonstrating that the expression of these proteins varied according to the genotype, growing location, season, and sowing time, with early cultivation showing lower levels of anti-nutritional factors [34]. However, more studies are required to explore the potential health benefits and risks associated with these factors.

Another aspect of studying the proteomic profile of soybean bran is to assess the presence of allergenic proteins. When considering the use of soybean in human food, soy is one of the main food allergens and the group of proteins to be considered comprises the inflammation-resolution enzymes, lipoxygenases (LOX) (Figure 2). While LOX plays crucial roles in the biosynthesis of bioactive compounds and defense responses in soybean plants, their activity can also lead to the development of off-flavors in soybean oil and affect the functional properties of soy proteins [35,36,37]. In plants, these enzymes play an important physiological role because the hydroperoxidation of linoleic acid is the first step in the biosynthesis of substances that regulate the growth, the factors involved in wound healing, and the control of chronic-disease-related inflammation [35]. Lipoxygenases have numerous potential applications in the food industry, but the use of soybean seeds as food ingredients has sometimes been limited, particularly in Western cultures, due to their “grassy/beany” flavor [36], resulting from the enzymatic oxidation of linoleic acid and linolenic acid by lipoxygenases. Some consumers also prefer a more neutral flavor in soybean products [37]. Although our results show only two lipoxygenases in the coatless seeds and defatted bran, with lower levels in the latter, these proteins are still a focus for the industry, which continuously searches for ways to reduce or completely remove the amount of these proteins in soy-derived products to improve taste and acceptance and expand the consumer market [38]. Nevertheless, in the context of human food LOX can be a target to nutraceutical industry.

Besides that, in a previous study with these biological materials, among the 139 proteins, 19 presented parts with allergenic potential, but only seven (I1LXY1; I1JWK3; I1KDM8; C6TFC1; I1KPN3; O64458; C6SWW4) remained in the defatted bran after processing [39]. These proteins possess different amino acid sequences, molecular weights, and tertiary structures, contributing to their distinctive allergenic properties.

Global efforts have been made to develop hypoallergenic soybean varieties with diminished allergenic potential. Reducing the level of these proteins could be an interesting strategy to both reduce the allergenicity content and increase the scope of the use of the soybean cultivar BRS 537, and other genotypes in the human diet. Genetic engineering techniques, including gene silencing and protein modification, aim to decrease the expression or alter the structure of allergenic proteins. Thus, these approaches hold promise for the production of safer soy-based products. Moreover, continued research is needed to investigate the soybean allergenic proteins present in bran, as well as the impact of processing techniques on allergenicity.

## 4. Materials and Methods

### 4.1. Biological Material

The soybean cultivar BRS 537 was maintained in a greenhouse within optimal cultivation conditions until seed production. BRS 537 is an early conventional tropical cultivar with high yield potential and stability and was launched in 2020 [40]. To extract the protein, samples of mature seed without their seed coat and industrially processed defatted bran were collected in three biological repetitions. The biological materials were freshly collected and pulverized in a mortar with a pestle in liquid nitrogen before analysis.

The simulation of the industrial processing of soybeans to obtain further defatted bran was performed by the Food Science and Quality Center of ITAL (Institute of Food Technology of the Government of Sao Paulo). The process consisted of removing the tegument from mature soybean seeds, crushing the particles to homogenize them, and removing the oil using a solvent. The defatted bran was purified to remove solvent residues, and then underwent treatment with humid steam for 30 min and dry heat (60 °C) for 1 h.

To extract proteins, soybeans from three biological replicates were first ground into fine powder in a chilled mortar and pestle; about 3 g were then transferred to a 15-milliliter Falcon tube, followed by incubation with SDS-based lysis buffer (4% SDS, 50 mM DTT, 0.1% Tween 20, 100 mM Tris-HCl, pH 8.0) on a shaker for about 60 min. The samples were centrifuged at 4000× *g* for 20 min, and the supernatants were transferred to clean tubes. Acetone precipitation was performed by adding 6 × volumes of cold acetone followed by overnight incubation at −20 °C. The precipitates were collected by centrifugation at 16,000× *g* for 20 min at 4 °C and washed twice with ice-cold acetone. The resulting pellets were dissolved in 50 mM ammonium bicarbonate and subjected to overnight tryptic digestion at 37 °C. The digests were desalted using C18-based StageTips, as described previously [41], dried with a SpeedVac, and stored at −80 °C until further use.

### 4.2. LC–MS/MS Analysis

The LC–MS/MS analysis was performed using an Ultimate 3000 nanoLC coupled with a Q Exactive mass spectrometer (Thermo Fisher, Waltham, MA, USA) with minor modifications [42]. In brief, the peptides were resuspended in LC buffer A (0.1% formic acid in water) and loaded onto a trap column (PepMap C18, 2 cm × 100 μm, Thermo Fisher, USA) followed by separation on an in-house packed column (C18 ReproSil, 3.0 μm, 17 cm × 75 μm; Dr. Maisch, Ammerbuch, Germany). Survey scans (MS1) were acquired in a data-dependent-top-10 method with a resolution of 70,000, a scan range of 350–1700 Da, maximum injection time of 20 ms, and AGC target of $1\;\times \;10^{6}$. The MS/MS scans were obtained via higher-energy collisional dissociation (HCD) fragmentation with a resolution of 17,500, target value of ${5\;\times \;10}^{5}$, and maximum injection time of 100 ms.

A soybean-protein database (85,142 sequences) downloaded from UniProt Knowledge base was used for the database search. The search parameters included trypsin as the enzyme, with a maximum of two missed cleavage sites allowed; mass tolerances of 10 ppm and 20 ppm for precursor and fragments, respectively; protein N-terminal acetylation and methionine oxidation as variable modifications; cysteine-carbamide methylation as a fixed modification; peptide length of at least seven amino acids; and a false-discovery rate (FDR) of 1% for proteins.

### 4.3. Identification of Differentially Expressed Proteins

The output of the LC–MS/MS analysis was normalized and quantified based on Scaffold Software instructions, including protein size and sample depth as variables [43]. The differential expression in Log2 fold change was performed using Benjamin–Hochberg correction of *p*-values and FDR values using EdgeR software v.3.32.136 [44]. Only proteins with ≥2 unique peptides using peptide spectrum matches (PSMs) were retrieved [45].

The annotation of proteins was performed using Phytozome v13 software (https://phytozome-next.jgi.doe.gov/ accessed on 29 May 2022) and the Persephone genome browser (https://web.persephonesoft.com/ accessed on 29 May 2022). The annotation of the biological function of proteins was analyzed using the Wm82.a2 and Wm82.a4 reference genomes of soybean.

### 4.4. Systems Biology

The enrichment was performed using differentially expressed protein datasets via KEGG pathways and Phytozome databases (https://www.genome.jp/kegg/pathway.html accessed on 10 June 2022). Pathways with 3 or more representative proteins were considered enriched according to ShinyGO software (http://bioinformatics.sdstate.edu/go/ accessed on 10 June 2022). The amino acid composition was examined using ProtParam software (https://web.expasy.org/protparam/ accessed on 15 June 2022). A threshold of more than 10% essential amino acids in the protein composition was applied.

## 5. Conclusions

To our knowledge, this is the first work to describe and provide the global protein profiles of coatless seeds and processed soybean defatted bran from the elite tropical cultivar, BRS 537 (Embrapa, Brazil). The global proteomics analysis allowed a better understanding of the composition and function of this elite soybean. In addition, the protein panel presented here will allow researchers to develop new strategies to improve the nutritional quality of soybean products or improve other soybean genotypes. In addition, through a variety of biotechnological tools that are currently available, it is possible to remove unwanted metabolites and increase the number of those that are beneficial, adding value to the use of soybeans in human food, as well as in the animal-feed market. Moreover, these proteins could be targets for several synthetic biology strategies aiming to develop high quality protein contents, such as providing insights for the development of synthetic mimetic plant organelles for protein storage. Thus, by combining comparative proteomics with other omics tools and genome editing, researchers can identify specific genes that may be targeted for cultivar improvement.

For the first time, the soybean–bran proteome was explored and, as a result, a catalogue of proteins that tolerate industrial processes was generated. Soybean bran is often considered a waste product in soy processing. Understanding the proteomic changes that occur in soybean bran after industrial processing provides insights about the utilization strategies for this by-product. By identifying proteins that are resistant to degradation or with unique functional properties, researchers can explore new methods for using soy bran in various food formulations. Thus, understanding the protein profile in soybean bran can orient the development of sustainable and value-added industrial application strategies for soybean bran.

## Data Availability

Data are available on request from the authors.

## Supplementary Materials

The following supporting information can be downloaded at: https://www.mdpi.com/article/10.3390/plants12142704/s1. The Supplementary File S1 can be accessed at https://github.com/Rech-PBSyn/Soybean-Proteome/ accessed on 14 July 2023. The raw data are available on the Mass Spectrometry Interactive Virtual Environment-MassIVE proteomic server (MSV000086419; https://massive.ucsd.edu/ProteoSAFe/static/massive-quant.jsp/ accessed on 29 May 2022).
